# A Local Electricity and Carbon Trading Method for Multi-Energy Microgrids Considering Cross-Chain Interaction

**DOI:** 10.3390/s22186935

**Published:** 2022-09-14

**Authors:** Xiaoqing Zhong, Yi Liu, Kan Xie, Shengli Xie

**Affiliations:** 1 School of Automation, Guangdong University of Technology, Guangzhou 510006, China; 2 Key Laboratory of Intelligent Information Processing and System Integration of IoT, Ministry of Education, Guangzhou 510006, China; 3 111 Center for Intelligent Batch Manufacturing Based on IoT Technology, Guangzhou 510006, China; 4 Guangdong Key Laboratory of IoT Information Technology, Guangzhou 510006, China; 5 Guangdong-HongKong-Macao Joint Laboratory for Smart Discrete Manufacturing, Guangzhou 510006, China

**Keywords:** multi-energy microgrid, electricity and carbon trading, Nash bargaining, blockchain, cross-chain interaction

## Abstract

The objective of this paper is to propose a local electricity and carbon trading method for interconnected multi-energy microgrids. A local electricity market and a local carbon market are established, allowing microgrids to trade electricity and carbon allowance within the microgrid network. Specifically, excessive electricity and carbon allowance of a microgrid can be shared with other microgrids that require them. A local electricity trading problem and a local carbon trading problem are formulated for multi-energy microgrids using the Nash bargaining theory. Each Nash bargaining problem can be decomposed into two subproblems, including an energy/carbon scheduling problem and a payment bargaining problem. By solving the subproblems of the Nash bargaining problems, the traded amounts of electricity/carbon allowance between microgrids and the corresponding payments will be determined. In addition, to enable secure information interactions and trading payments, we introduce an electricity blockchain and a carbon blockchain to record the trading data for microgrids. The novelty of the usage of the blockchain technology lies in using a notary mechanism-based cross-chain interaction method to achieve value transfer between blockchains. The simulation results show that the proposed local electricity and carbon trading method has great performance in lowering total payments and carbon emissions for microgrids.

## 1. Introduction

With the benefits of promoting a sustainable society and reducing carbon emissions, many countries greatly support the use of renewable energy to replace traditional fossil fuels [1]. Installing distributed power sources, e.g., photovoltaic panels and wind turbines, in microgrids will provide many advantages, such as reducing energy procurement costs and lowering carbon emissions by consuming renewable energy [2]. To reduce the curtailment of renewable energy and long-distance electricity transmission, the local electricity trading mechanism is introduced into the power market, which will allow microgrids to become prosumers that can buy and sell electricity in the local electricity market [3]. Through local electricity trading, microgrids will have an opportunity to obtain revenues by selling their excess renewable energy. Therefore, local electricity trading provides economic and environmental benefits for microgrids [4].

Considering that microgrids are both energy consumers and carbon emitters, the inclusion of microgrids in the electricity and carbon markets is necessary [5]. With the construction of electricity transmission infrastructure between microgrids, local electricity trading can be conducted within the microgrid network. In addition, microgrids should pay for carbon emissions. Introducing the carbon market, e.g., the carbon cap-and-trade market [6], is an effective way to impact the carbon emissions from microgrids. When the carbon emissions of microgrids exceed their allocated carbon allowance, they should purchase the deficient carbon allowance, which will motivate them to reduce carbon emissions.

Many researchers, e.g., [7,8,9,10], have investigated electricity or carbon trading between entities. In [7], a Nash-bargaining-based electricity interaction method is proposed for interconnected microgrids, and the alternating direction method of multipliers (ADMM) method is used to solve Nash bargaining problems in a distributed manner. An optimal energy management method for multi-energy multi-microgrid networks is proposed in [8], where two scheduling horizons including the day-ahead phase and intra-day phase are considered. An investment decision model for microgrids is proposed in [9], where the impacts of the carbon trading mechanism are taken into account. In [10], a dynamic economic dispatch model of plug-in electric vehicles is constructed, and the effectiveness of compensating battery degradation costs with carbon trading revenues is verified. Furthermore, some studies, e.g., [5,11], consider both electricity and carbon trading. A bi-level distributed day-ahead schedule model for multi-microgrids is proposed in [11], where both the local electricity trading and local carbon trading between microgrids are taken into account. A cooperative operation method for a battery swapping station, a charging station, and a residential building is proposed in [5], where the electricity and carbon trading between these three entities is considered. However, these papers [5,7,8,9,10,11] have not considered that when entities operate cooperatively, the payments for local electricity trading and local carbon trading may be separate.

Blockchain is an immutable and distributed ledger, which provides a secure way to record transaction data [12]. Many researchers, e.g., [13,14], have considered using the blockchain technology to establish a secure local electricity market. Ref. [13] proposes a decentralized peer-to-peer (P2P) energy trading scheme that performs via blockchain-based smart contracts. Ref. [14] considers a blockchain-based P2P energy trading system between prosumers and consumers. Furthermore, some studies, e.g., [15,16], have considered using a blockchain to store transaction data for both electricity and carbon markets. In [15], electricity and carbon trading markets are established for networked microgrids, and a blockchain is applied to save the transaction data associated with electricity and carbon trading. A fully decentralized blockchain-based P2P trading scheme for prosumers is proposed in [16], where a blockchain is introduced to record both electricity and carbon trading data. However, these papers [13,14,15,16] have not considered that there would be two different blockchains, one for the electricity market and the other for the carbon market.

In this paper, a local electricity and carbon trading method for multi-energy microgrids is proposed using the Nash bargaining theory [7]. The local electricity and carbon markets are established within the microgrid network. An electricity blockchain is introduced to record the electricity trading data and a carbon blockchain is introduced to record carbon trading data. Two cryptocurrencies, named electricity coins and carbon coins, are used to trade payments in the electricity blockchain and carbon blockchain, respectively. Furthermore, a notary mechanism-based cross-chain interaction method is proposed to realize value transfer between electricity and carbon blockchains [17,18].

The contributions of this paper are summarized below:

1. We propose a Nash bargaining-based local electricity and carbon trading method for interconnected multi-energy microgrids, which helps to determine the traded amounts of electricity and carbon allowance between microgrids and the corresponding local electricity and carbon payments of microgrids in a fair manner;
2. We introduce an electricity blockchain and a carbon blockchain for secure information interactions and payments, while electricity coins and carbon coins are introduced as cryptocurrencies for these two blockchains, respectively;
3. A notary mechanism-based cross-chain interaction method is proposed to achieve value transfer between the electricity and carbon blockchains.

The rest of this paper is structured in the following manner: Section 2 describes the system model of the microgrid and microgrid network. Section 3 develops the mathematical formulation of the local electricity and carbon trading problems. Section 4 introduces the electricity and carbon blockchains and presents the cross-chain interaction method. Case studies are conducted in Section 5. Finally, we conclude this paper in Section 6.

## 2. System Model

In this section, we will first present the basic structures of multi-energy microgrids and their connective topology. Then, the detailed models of various energy devices and the operation constraints of microgrids are given.

### 2.1. Microgrid Model

**Figure 1:**
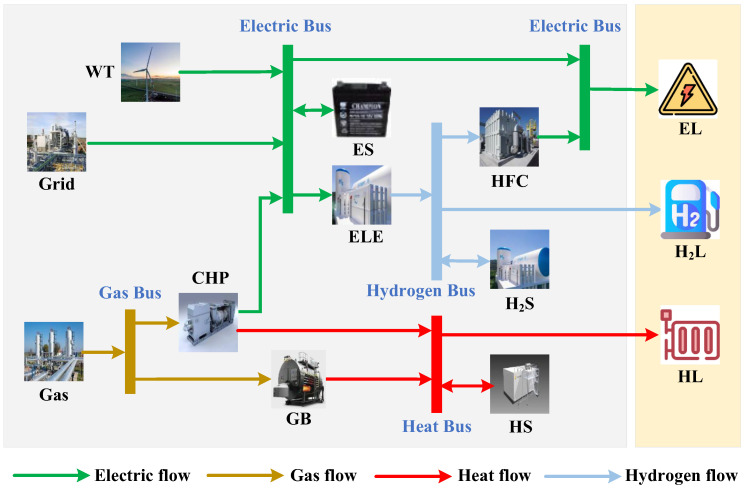
The basic structure of a multi-energy microgrid.

Figure 1 shows the basic structure of a multi-energy microgrid. Each microgrid i∈M contains the following components: a wind turbine (WT), a combined heat and power (CHP) unit, a gas boiler (GB), an electrolyzer (ELE), a hydrogen fuel cell (HFC), electric storage (ES), heat storage (HS), and hydrogen storage (H_2_S). Three different types of load demands for microgrids should be met: electric loads (ELs), heat loads (HLs), and hydrogen loads (H_2_Ls). Microgrids will participate in both local electricity and carbon markets. Thus, each microgrid can sell its excess renewable energy to other microgrids that need to purchase electricity. In the meantime, the carbon allowance of microgrids can be traded within the microgrid network.

### 2.2. Microgrid Network

**Figure 2:**
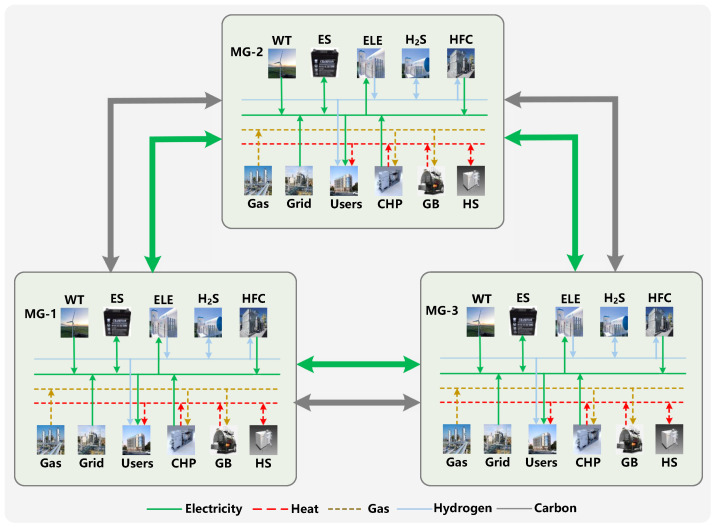
The schematic model of the microgrid network (MG: microgrid).

Figure 2 presents the schematic model of the microgrid network that is composed of three microgrids (i.e., MG-1, MG-2, and MG-3). Let M={1,…,M} denote the set of *M* microgrids. Microgrids can purchase electricity and gas from the electric and gas utilities. In the meantime, microgrids can purchase insufficient or sell surplus carbon allowance in the central carbon market. Microgrids are connected to each other through electricity transmission infrastructures, which enables local electricity trading within the microgrid network. Moreover, microgrids are connected to each other through the wireless communication network, which realizes the information transmission between microgrids [19,20]. The operation horizon T={1,…T} is composed of *T* equal time slots, and each time slot is denoted as t∈T. A microgrid manager is responsible for scheduling energy and carbon allowance and clearing local electricity and carbon markets for microgrids. Note that the carbon trading can be conducted not only in a global market or national market, but also in the local markets. Many researchers, e.g., [10,15,21], have focused on the ability of local carbon markets and entities in the markets to trade carbon allowance with each other. Specifically, entities participating in local carbon markets can include microgrids [15], energy hubs [21], and plug-in electric vehicles [10], etc. Thus, we believe that it is reasonable to integrate multi-energy microgrids into the local carbon market. Moreover, the local carbon market considered in this paper is similar to the global/national carbon markets. The carbon emissions of a multi-energy microgrid can be calculated as Equation (23). Then, each microgrid can sell its excess carbon allowance to other microgrids that need to purchase carbon allowance. Thus, we believe that the local carbon trading between microgrids can emulate the global/national carbon markets.

### 2.3. Microgrid Operation Constraints

The detailed models of energy devices and the operation constraints of microgrids are formulated as follows.

(1) Power sources: Each microgrid can purchase electricity and gas from utilities, and the wind turbine will supply renewable energy for the microgrid. Let $P_{t,i}^{GRID}$ and $P_{t,i}^{GAS}$ denote the amounts of electricity and gas purchased from the utilities, respectively; let $P_{t,i}^{WT}$ denote the scheduled wind power. We have:

(1) $$0\leq P_{t,i}^{GRID}\leq P_{i,\max}^{GRID},$$

(2) $$0\leq P_{t,i}^{GAS}\leq P_{i,\max}^{GAS},$$

(3) $$0\leq P_{t,i}^{WT}\leq P_{t,i}^{WT,gen},$$

where $P_{i,\max}^{GRID}$ and $P_{i,\max}^{GAS}$ denote the maximum amounts of electricity and gas that microgrid *i* can purchase from the electric and gas utilities, respectively, and $P_{t,i}^{WT,gen}$ denotes the wind power output of microgrid *i* in time slot *t*.

(2) CHP: Let $P_{t,i}^{CHP,g}$ denote the gas consumption of the CHP. Its electricity and heat outputs are defined as $P_{t,i}^{CHP,e}$ and $P_{t,i}^{CHP,h}$, respectively. The operation constraints of the CHP can be described by:

(4) $$P_{t,i}^{CHP,e}=\eta_{i}^{CHP,e}P_{t,i}^{CHP,g},$$

(5) $$P_{t,i}^{CHP,h}=\eta_{i}^{CHP,h}P_{t,i}^{CHP,g},$$

(6) $$0\leq P_{t,i}^{CHP,g}\leq P_{i,\max}^{CHP,g},$$

(7) $$R_{i}^{CHP,d}\leq \left(P_{t,i}^{CHP,g}-P_{t-1,i}^{CHP,g}\right)/\Delta t\leq R_{i}^{CHP,u},$$

where $\eta_{i}^{CHP,e}$ and $\eta_{i}^{CHP,h}$ represent electricity and heat conversion efficiencies of the CHP, respectively; $P_{i,\max}^{CHP,g}$ denotes the maximum gas consumption of the CHP; $R_{i}^{CHP,u}$ and $R_{i}^{CHP,d}$ denote the ramp up/down limits of the CHP, respectively; and Δt represents a time slot (i.e., an hour in this paper).

(3) GB: The GB can consume gas to satisfy the heat demands of the microgrid. Let $P_{t,i}^{GB,g}$ denote the gas consumption of the GB. Its heat output is denoted as $P_{t,i}^{GB,h}$. The GB should satisfy the following constraints:

(8) $$P_{t,i}^{GB,h}=\eta_{i}^{GB,h}P_{t,i}^{GB,g},$$

(9) $$0\leq P_{t,i}^{GB,g}\leq P_{i,\max}^{GB,g},$$

where $\eta_{i}^{GB,h}$ denotes the heat conversion efficiency of the GB; $P_{i,\max}^{GB,g}$ denotes the maximum gas consumption of the GB.

(4) ELE: The ELE can generate hydrogen by consuming electricity. Let $P_{t,i}^{ELE,e}$ denote the electricity consumption of the ELE. Its hydrogen output is denoted as $P_{t,i}^{ELE,h_{2}}$. The operation constraints of the ELE are given as:

(10) $$P_{t,i}^{ELE,h_{2}}=\eta_{i}^{ELE,h_{2}}P_{t,i}^{ELE,e},$$

(11) $$0\leq P_{t,i}^{ELE,e}\leq P_{i,\max}^{ELE,e},$$

where $\eta_{i}^{ELE,h_{2}}$ denotes the hydrogen conversion efficiency of the ELE; $P_{i,\max}^{ELE,e}$ denotes the maximum electricity consumption of the ELE.

(5) HFC: The HFC can generate electricity by consuming hydrogen. Let $P_{t,i}^{HFC,h_{2}}$ denote the hydrogen consumption of the HFC. Its electricity output is denoted as $P_{t,i}^{HFC,e}$. The operation constraints of the HFC are given as:

(12) $$P_{t,i}^{HFC,e}=\eta_{i}^{HFC,e}P_{t,i}^{HFC,h_{2}},$$

(13) $$0\leq P_{t,i}^{HFC,h_{2}}\leq P_{i,\max}^{HFC,h_{2}},$$

where $\eta_{i}^{HFC,e}$ denotes the electricity conversion efficiency of the HFC and $P_{i,\max}^{HFC,h_{2}}$ represents the maximum hydrogen consumption of the HFC.

(6) STO: We define $\mathrm{STO}\in S\overset{\Delta }{\;=}\{\mathrm{ES},\mathrm{HS},H_{2}S\}$ as a type of storage. The ES, HS, and H_2_S have similar operation constraints. Let $E_{t,i}^{STO}$ denote the energy level of the STO; let $P_{t,i}^{STO,c}$ and $P_{t,i}^{STO,d}$ denote the charging and discharging power of the STO, respectively; let $\eta_{i}^{STO,c}$ and $\eta_{i}^{STO,d}$ denote the charging and discharging efficiencies of the STO, respectively. The operation constraints of the STO can be described by:

(14) $$E_{t,i}^{STO}=E_{t-1,i}^{STO}+\eta_{i}^{STO,c}P_{t,i}^{STO,c}\Delta t-\left(1/\eta_{i}^{STO,d}\right)P_{t,i}^{STO,d}\Delta t,$$

(15) $$E_{i,\min}^{STO}\leq E_{t,i}^{STO}\leq E_{i,\max}^{STO},$$

(16) $$0\leq P_{t,i}^{STO,c}\leq P_{i,\max}^{STO,c},$$

(17) $$0\leq P_{t,i}^{STO,d}\leq P_{i,\max}^{STO,d},$$

(18) $$E_{0,i}^{STO}=E_{T,i}^{STO},$$

where $E_{i,\max}^{STO}$ and $E_{i,\min}^{STO}$ represent the maximum and minimum energy levels of the STO, respectively; $P_{i,\max}^{STO.c}$ and $P_{i,\max}^{STO,d}$ represent the maximum charging and discharging power of the STO, respectively; $E_{0,i}^{STO}$ and $E_{T,i}^{STO}$ denote the energy levels of the STO in the initial and terminal time slots, respectively. Note that constraint (18) denotes that the energy levels of the STO are equal in the initial and terminal time slots [22].

(7) Power balance equations: The electricity, heat, hydrogen, and gas balance equations of microgrid *i* are shown as:

(19) $$P_{t,i}^{GRID}+P_{t,i}^{WT}+P_{t,i}^{CHP,e}+P_{t,i}^{HFC,e}+P_{t,i}^{ES,d}+\sum_{j\in M\setminus i}P_{t,i}^{j}=P_{t,i}^{EL}+P_{t,i}^{ES,c}+P_{t,i}^{ELE,e},$$

(20) $$P_{t,i}^{CHP,h}+P_{t,i}^{GB,h}+P_{t,i}^{HS,d}=P_{t,i}^{HL}+P_{t,i}^{HS,c},$$

(21) $$P_{t,i}^{ELE,h_{2}}+P_{t,i}^{H_{2}S,d}=P_{t,i}^{H_{2}L}+P_{t,i}^{HFC,h_{2}}+P_{t,i}^{H_{2}S,c},$$

(22) $$P_{t,i}^{GAS}=P_{t,i}^{CHP,g}+P_{t,i}^{GB,g},$$

where $P_{t,i}^{j},\forall i,j\in M,i\neq j$ denotes the electricity that microgrid *i* trades with microgrid *j*. If microgrid *i* purchases electricity from microgrid *j*, $P_{t,i}^{j}$ is positive; otherwise, microgrid *i* sells electricity to microgrid *j*, and $P_{t,i}^{j}$ is negative.

(8) Carbon emissions and trading: Let $W_{i,t}^{CO_{2}}$ denote the carbon emissions of microgrid *i*, which can be expressed as [23]:

(23) $$\begin{array}{c} W_{i,t}^{CO_{2}}=em_{i}^{GRID}P_{t,i}^{GRID}+em_{i}^{CHP}P_{t,i}^{CHP,g}+em_{i}^{GB}P_{t,i}^{GB,g}+\\ \;\;\;\;em_{i}^{ELE}P_{t,i}^{ELE,e}+em_{i}^{HFC}P_{t,i}^{HFC,e}+em_{i}^{ES}\left(P_{t,i}^{ES,c}+P_{t,i}^{ES,d}\right) \end{array},$$

where $em_{i}^{CHP}$, $em_{i}^{GB}$, $em_{i}^{ELE}$, $em_{i}^{HFC}$ and $em_{i}^{ES}$ denote the carbon emission factors of CHP, GB, ELE, HFC, and ES, respectively; $em_{i}^{GRID}$ denotes the carbon emission factor associated with the electricity procurement from the electric utility. In the carbon cap-and-trade market, microgrids will be allocated certain amounts of carbon allowance for free. When microgrids purchase or sell carbon allowance in the central carbon market, they should satisfy:

(24) $$0\leq W_{i}^{CO_{2},buy}\leq W_{i,\max}^{CO_{2},buy},$$

(25) $$0\leq W_{i}^{CO_{2},sell}\leq W_{i,\max}^{CO_{2},sell},$$

where $W_{i,\max}^{CO_{2},buy}$ and $W_{i,\max}^{CO_{2},sell}$ denote the maximum amounts of carbon allowance that microgrid *i* can purchase or sell in the central carbon market during one day.

(9) Carbon balance equation: The carbon balance equation means that the total amount of carbon allowance owned by microgrid *i* is equal to its total carbon emissions, which can be formulated as:

(26) $$W_{i}^{CO_{2},a}+W_{i}^{CO_{2},buy}+\sum_{j\in M\setminus i}W_{i}^{CO_{2},j}=\sum_{t\in T}W_{i,t}^{CO_{2}}+W_{i}^{CO_{2},sell},$$

where $W_{i}^{CO_{2},a}$ represents the allocated carbon allowance of microgrid *i*; and $W_{i}^{CO_{2},j},\forall i,j\in M,i\neq j$ denotes the amount of carbon allowance that microgrid *i* trades with microgrid *j*. If microgrid *i* purchases carbon allowance from microgrid *j*, $W_{i}^{CO_{2},j}$ is positive; otherwise, microgrid *i* sells carbon allowance to microgrid *j*, and $W_{i}^{CO_{2},j}$ is negative.

(10) Local market clearing constraints: The local electricity and carbon trading must satisfy the following market clearing constraints:

(27) $$P_{t,i}^{j}+P_{t,j}^{i}=0,$$

(28) $$W_{i}^{CO_{2},j}+W_{j}^{CO_{2},i}=0,$$

Note that due to the close distances between microgrids, we neglect the energy losses during the electricity transmission.

## 3. Local Electricity and Carbon Trading

In this section, we present the mathematical formulations of the local electricity trading problem and local carbon trading problem. Since the scheduling horizons and market mechanisms of the energy market and carbon market are different, we consider that the local electricity trading and local carbon trading are conducted sequentially. Specifically, the local electricity trading is conducted in each time slot t∈T and the local carbon trading is conducted after the energy scheduling, i.e., after t=24.

### 3.1. Non-Cooperative Benchmarks

In the non-cooperative benchmarks, microgrids will not exchange electricity and carbon allowance with each other, i.e., the local electricity and carbon trading will not be conducted. Thus, the electricity and carbon balance equations of microgrid *i* should be revised as:

(29) $$P_{t,i}^{GRID}+P_{t,i}^{WT}+P_{t,i}^{CHP,e}+P_{t,i}^{HFC,e}+P_{t,i}^{ES,d}=P_{t,i}^{EL}+P_{t,i}^{ES,c}+P_{t,i}^{ELE,e},$$

(30) $$W_{i}^{CO_{2},a}+W_{i}^{CO_{2},buy}=\sum_{t\in T}W_{i,t}^{CO_{2}}+W_{i}^{CO_{2},sell},$$

For the local electricity trading problem, the objective of each microgrid is to minimize its operation cost $F_{i}^{OPE}$, which consists of electricity purchase cost $F_{i}^{GRID}$, gas purchase cost $F_{i}^{GAS}$, and storage degradation cost $F_{i}^{S,\deg}$. The operation cost of microgrid *i* is given as:

(31) $$F_{i}^{OPE}=F_{i}^{GRID}+F_{i}^{GAS}+F_{i}^{S,\deg},$$

(32) $$F_{i}^{GRID}=\sum_{t\in T}\left(\lambda_{t}^{e}P_{t,i}^{GRID}\Delta t\right),$$

(33) $$F_{i}^{GAS}=\sum_{t\in T}\left(\lambda_{t}^{g}P_{t,i}^{GAS}\Delta t\right),$$

(34) $$F_{i}^{S,\deg}=\sum_{STO\in S}\sum_{t\in T}\lambda_{i}^{STO,\deg}\left(P_{t,i}^{STO,c}+P_{t,i}^{STO,d}\right),$$

where $\lambda_{t}^{e}$ and $\lambda_{t}^{g}$ denote the electricity and gas prices of utilities; $\lambda_{i}^{STO,\deg}$ denotes the amortized cost of the charging and discharging of the STO. Let **P1-NC${}_{i}$** denote the operation cost minimization problem of microgrid *i* in the non-cooperative benchmarks. We have:

**P1-NC**${}_{i}$:

$$\begin{array}{c} \min_{\Phi_{NC_{1}}}F_{i}^{OPE}, \end{array}$$

subject to: (1)–(18), (20)–(22), and (29),

where decision variables $\Phi_{NC_{1}}=\{P_{t,i}^{GRID},P_{t,i}^{GAS},P_{t,i}^{WT},P_{t,i}^{CHP,g},P_{t,i}^{GB,g},P_{t,i}^{ELE,e},P_{t,i}^{HFC,h_{2}},E_{t,i}^{STO},$$P_{t,i}^{STO,c},P_{t,i}^{STO,d}\left|t\in T,i\in M,STO\in S\right\}$. Here, we define $\left\{F_{i}^{NC,e}|i\in M\right\}$ as the optimal objective function value of the **P1-NC${}_{i}$**.

Let $F_{i}^{CE}$ denote the total carbon cost of microgrid *i*. We have:

(35) $$F_{i}^{CE}=\beta^{b}W_{i}^{CO_{2},buy}-\beta^{s}W_{i}^{CO_{2},sell},$$

where $\beta^{b}$ and $\beta^{s}$ denote the buying and selling prices of carbon allowance in the central carbon market, respectively. Let **P2-NC${}_{i}$** denote the carbon cost minimization problem of microgrid *i* in the non-cooperative benchmarks. We have:

**P2-NC**${}_{i}$:

$$\begin{array}{c} \min_{\Phi_{NC_{2}}}F_{i}^{CE}, \end{array}$$

subject to: (24), (25), and (30),

where decision variables $\Phi_{NC_{2}}=\left\{W_{i}^{CO_{2},buy},W_{i}^{CO_{2},sell}|i\in M\right\}$. We define $\left\{F_{i}^{NC,c}|i\in M\right\}$ as the optimal objective function value of the **P2-NC${}_{i}$**.

### 3.2. Local Trading Payments

Microgrids should pay for the electricity and carbon allowance that are traded in the local markets. Let $\pi_{i}^{j}$ and $\gamma_{i}^{j}$ denote the payments associated with the electricity and carbon trading between microgrids *i* and *j*, respectively. If microgrid *i* makes payments to microgrid *j*, $\pi_{i}^{j}$ and $\gamma_{i}^{j}$ will be positive; otherwise, microgrid *i* receives payments from microgrid *j*, $\pi_{i}^{j}$ and $\gamma_{i}^{j}$ will be negative. The following payment balance equations should be satisfied:

(36) $$\pi_{i}^{j}+\pi_{j}^{i}=0,$$

(37) $$\gamma_{i}^{j}+\gamma_{j}^{i}=0,$$

Let $F_{i}^{PAY,e}$ and $F_{i}^{PAY,c}$ denote the total payments of microgrid *i* for local electricity trading and local carbon trading, respectively. We have:

(38) $$F_{i}^{PAY,e}=\sum_{j\in M\setminus i}\pi_{i}^{j},$$

(39) $$F_{i}^{PAY,c}=\sum_{j\in M\setminus i}\gamma_{i}^{j},$$

A microgrid will only participate in the local electricity and carbon trading if it can reduce the total payment for the microgrid. Thus, we have:

(40) $$F_{i}^{OPE}+F_{i}^{PAY,e}\leq F_{i}^{NC,e},$$

(41) $$F_{i}^{CE}+F_{i}^{PAY,c}\leq F_{i}^{NC,c},$$

### 3.3. Nash Bargaining Problems

We denote the Nash bargaining problems of the local electricity trading and local carbon trading as **P1-NB** and **P2-NB**, respectively. The **P1-NB** is shown as follows:

**P1-NB:**

$$\begin{array}{c} \max_{\Phi_{NB_{1}}}\underset{i\in M}{\Pi }\left[F_{i}^{NC,e}-\left(F_{i}^{OPE}+F_{i}^{PAY,e}\right)\right], \end{array}$$

subject to: (1)–(22), (27), (36), and (40),

where decision variables $\Phi_{NB_{1}}=\{P_{t,i}^{GRID},P_{t,i}^{GAS},P_{t,i}^{WT},P_{t,i}^{CHP,g},P_{t,i}^{GB,g},P_{t,i}^{ELE,e},P_{t,i}^{HFC,h_{2}},E_{t,i}^{STO},$$P_{t,i}^{STO,c},P_{t,i}^{STO,d},P_{t,i}^{j},\pi_{i}^{j}\left|t\in T,i\in M,j\in M\setminus i,STO\in S\right\}$. By solving the **P1-NB**, the energy scheduling results and the local electricity trading payments will be obtained for microgrids. Note that based on the energy-scheduling results, the carbon emissions of microgrids can be calculated by (23).

The **P2-NB** is presented as follows:

**P2-NB:**

$$\begin{array}{c} \max_{\Phi_{NB_{2}}}\underset{i\in M}{\Pi }\left[F_{i}^{NC,c}-\left(F_{i}^{CE}+F_{i}^{PAY,c}\right)\right], \end{array}$$

subject to: (24)–(26), (28), (37), and (41),

where decision variables $\Phi_{NB_{2}}=\{W_{i}^{CO_{2},buy},W_{i}^{CO_{2},sell},W_{i}^{CO_{2},j},\gamma_{i}^{j}\left|i\in M,j\in M\setminus i\right\}$. By solving the **P2-NB**, the carbon allowance scheduling results and the local carbon trading payments will be obtained for microgrids.

### 3.4. Solution Method

The Nash bargaining problems **P1-NB** and **P2-NB** are non-convex, as they have non-convex objective functions. Specifically, the **P1-NB** will maximize the differences between disagreement point $F_{i}^{NC,e}$ and total operation cost $\left(F_{i}^{OPE}+F_{i}^{PAY,e}\right)$ of microgrid *i* through bargaining, while the **P2-NB** will maximize the differences between disagreement point $F_{i}^{NC,c}$ and total carbon cost $\left(F_{i}^{CE}+F_{i}^{PAY,c}\right)$ through bargaining. The product formulations in the objective functions will guarantee that the benefits of cooperation can be fairly distributed by microgrids that participate in the local electricity and carbon markets [7]. Based on the findings of the previous research [5], the Nash bargaining problems **P1-NB** and **P2-NB** can be solved via decomposition into two subproblems, respectively.

In this paper, the **P1-NB** is decomposed into an energy-scheduling problem **S1-ET** and an electricity payment bargaining problem **S2-EP**. The **P2-NB** is decomposed into a carbon scheduling problem **S1-CT** and a carbon payment bargaining problem **S2-CP**. The **S1-ET** and **S2-EP** are presented as follows:

**S1-ET:**

$$\begin{array}{c} \min_{\Phi_{{}_{ET}}}\sum_{i\in M}F_{i}^{OPE}, \end{array}$$

subject to: (1)–(22), and (27),

where decision variables $\Phi_{ET}=\{P_{t,i}^{GRID},P_{t,i}^{GAS},P_{t,i}^{WT},P_{t,i}^{CHP,g},P_{t,i}^{GB,g},P_{t,i}^{ELE,e},P_{t,i}^{HFC,h_{2}},E_{t,i}^{STO},$$P_{t,i}^{STO,c},P_{t,i}^{STO,d},P_{t,i}^{j}\left|t\in T,i\in M,j\in M\setminus i,STO\in S\right\}$. Solving the **S1-ET** determines the energy scheduling and trading results for each microgrid i∈M.

**S2-EP:**

$$\begin{array}{c} \max_{\Phi_{{}_{EP}}}\underset{i\in M}{\Pi }\left[F_{i}^{NC,e}-\left({\bar{F}}_{i}^{OPE}+F_{i}^{PAY,e}\right)\right], \end{array}$$

subject to: (36) and (40),

where decision variables $\Phi_{EP}=\{\pi_{i}^{j}\left|i\in M,j\in M\setminus i\right\}$; and ${\bar{F}}_{i}^{OPE}$ denotes the optimal operation cost of microgrid *i*, which is obtained by solving the **S1-ET**. Solving the **S2-EP** determines the electricity trading payments for microgrids.

Now, we present the **S1-CT** and **S2-CP** as follows:

                     **S1-CT:**

$$\begin{array}{c} \min_{\Phi_{{}_{CT}}}\sum_{i\in M}F_{i}^{CE}, \end{array}$$

subject to: (24)–(26), and (28),

where decision variables $\Phi_{CT}=\{W_{i}^{CO_{2},buy},W_{i}^{CO_{2},sell},W_{i}^{CO_{2},j}\left|i\in M,j\in M\setminus i\right\}$. The carbon scheduling and trading results of microgrids will be obtained by solving the **S1-CT**.

**S2-CP:**

$$\begin{array}{c} \max_{\Phi_{{}_{CP}}}\underset{i\in M}{\Pi }\left[F_{i}^{NC,c}-\left({\bar{F}}_{i}^{CE}+F_{i}^{PAY,c}\right)\right], \end{array}$$

subject to: (37) and (41),

where decision variables $\Phi_{CP}=\{\gamma_{i}^{j}\left|i\in M,j\in M\setminus i\right\}$; ${\bar{F}}_{i}^{CE}$ denotes the optimal carbon cost of microgrid *i*, which is obtained by solving the **S1-CT**. The carbon trading payments of microgrids will be obtained by solving the **S2-CP**.

The constraints and objective functions of the scheduling problems **S1-ET** and **S1-CT** are linear, which means that the **S1-ET** and **S1-CT** are both convex problems. We can obtain the optimal energy and carbon allowance scheduling results for microgrids. Thus, the non-convexity of the Nash bargaining problems will not prevent us from obtaining the optimal scheduling results for microgrids.

The non-convexity of the Nash bargaining problems **P1-NB** and **P2-NB** helps to arrange the usage of energy and carbon allowance and conduct payment bargaining. Solving the Nash bargaining problems can not only obtain the optimal energy and carbon allowance scheduling results, but also the corresponding local payments for microgrids. The corresponding local payments of microgrids will help to fairly distribute the profits obtained from cooperation among microgrids. Moreover, although the Nash bargaining problems **P1-NB** and **P2-NB** are non-convex, we can obtain the globally optimal solutions for these two problems. This is because each Nash bargaining problem can be solved by decomposing it into two convex subproblems. The optimal solutions of the subproblems can be found, and the solutions are the Nash bargaining solutions as well [5].

### 3.5. Solution Procedures

The **S1-ET** and **S1-CT** are convex problems, which can be easily solved by off-the-shelf solvers, e.g., Gurobi [24]. However, the **S2-EP** and **S2-CP** are non-convex problems. By taking the log and negating the objective functions of the **S2-EP** and **S2-CP**, we can rewrite the objective functions of these two problems as follows:

(42) $$\min_{\Phi_{{}_{EP}}}\sum_{i\in M}-\log(F_{i}^{NC,e}-\left({\bar{F}}_{i}^{OPE}+F_{i}^{PAY,e}\right)),$$

(43) $$\min_{\Phi_{{}_{CP}}}\sum_{i\in M}-\log(F_{i}^{NC,c}-\left({\bar{F}}_{i}^{CE}+F_{i}^{PAY,c}\right)),$$

Under the new objective functions, the **S2-EP** and **S2-CP** will become convex problems as well. We can solve these two problems using commercial solvers, e.g., Ipopt [25].

**Figure 3:**
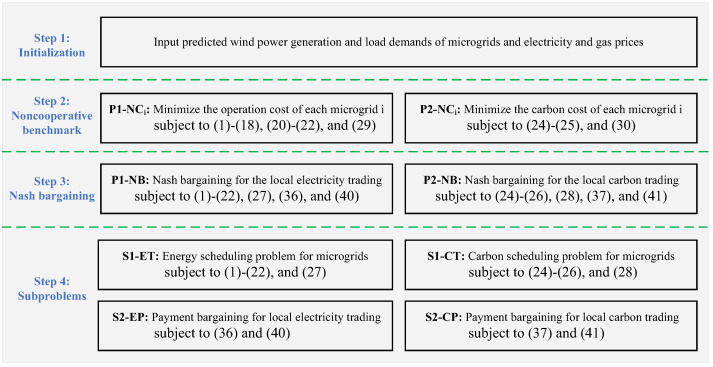
Implementations of the local electricity and carbon trading.

The implementations of the local electricity and carbon trading between microgrids are presented in Figure 3.

## 4. Cross-Chain Interaction Method

In this section, we first introduce the electricity and carbon blockchains. Then, a notary mechanism-based cross-chain interaction method is proposed to realize the value transfer between the electricity blockchain and the carbon blockchain.

### 4.1. Electricity and Carbon Blockchains

Two private blockchains, an electricity blockchain and a carbon blockchain, are considered in this paper. These two blockchains act as digital databases and store the metadata of local electricity and carbon trading information of microgrids. The microgrid manager is responsible for recording the trading information of microgrids in the electricity and carbon blockchains. The trading information includes pseudonyms of microgrids, transaction data, and a timestamp of transaction generation. The trading information will be structured into blocks, and the blocks are chronologically added into the blockchains. The information of the blocks can only be accessed by microgrids and the microgrid manager. Two different digital cryptocurrencies, named electricity coins and carbon coins, are introduced into the electricity blockchain and the carbon blockchain, respectively. The electricity coins and carbon coins are used as currencies in the local electricity market and local carbon market, respectively.

**Figure 4:**
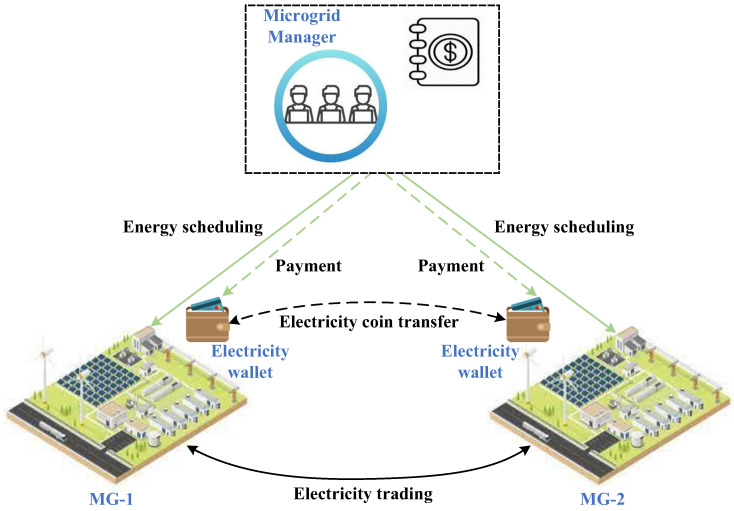
Local electricity trading and electricity coin transfer.

Here, we take the local electricity trading between microgrids as an example to illustrate the operation mechanism of the electricity blockchain. Firstly, the microgrid manager will solve the Nash bargaining problem of local electricity trading to obtain the local electricity payments for microgrids. Each microgrid has an electricity wallet to store its own electricity coins, which will be used to pay for the electricity obtained through local electricity trading. Then, the microgrid manager will implement the payments for microgrids, transferring the electricity coins between the electricity wallets of microgrids. Thus, the local electricity trading payments can be completed. The implementation of the local electricity trading and the corresponding electricity coin transfer are shown in Figure 4. Note that when a microgrid does not have enough electricity coins, it can exchange the fiat currency for the electricity coins via the microgrid manager. The microgrid manager will complete the exchanges in the cryptocurrency exchange center.

### 4.2. Cross-Chain Interactions

Since the electricity and carbon coins are used in the local electricity market and local carbon market, respectively, it is necessary to realize the exchange between these two types of cryptocurrencies. In this paper, a cross-chain interaction method is proposed to achieve the value transfer between the electricity blockchain and carbon blockchain. Through the cross-chain interactions, the electricity coins and carbon coins can be exchanged. In this way, the profits obtained through the local electricity/carbon trading can be used to cover the payments of the local carbon/electricity trading.

We use the notary mechanism to realize the cross-chain interaction. In the notary mechanism, a notary is introduced and is responsible for the cross-chain interactions. During the cross-chain interactions, the notary acts as a trusted third party to realize the exchange of electricity coins and carbon coins and truly record the exchange results in the blockchains. Based on the cryptocurrency exchange requests, the notary will achieve cryptocurrency exchange in the cryptocurrency exchange center. In this paper, we consider that the microgrid manager will act as the notary.

**Figure 5:**
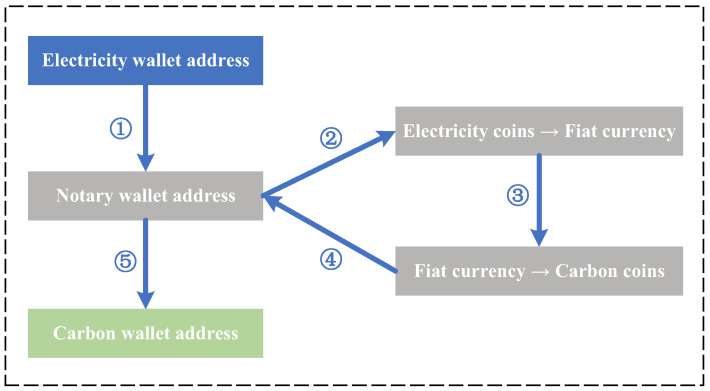
The processes of exchanging electricity coins into carbon coins.

Here, we take the exchange of electricity coins into carbon coins as an example to illustrate the processes of a cross-chain interaction. Figure 5 shows the processes of exchanging the electricity coins into carbon coins. The detailed steps of the cross-chain interaction are shown as follows:

1. Microgrid *i* transfers electricity coins from its electricity wallet to the wallet of the notary;
2. The notary receives the cryptocurrency exchange request and transfers the received electricity coins to the cryptocurrency exchange center;
3. The notary exchanges the electricity coins into the carbon coins based on the exchange rate between the electricity coins and carbon coins;
4. The exchanged carbon coins are transferred into the wallet of the notary;
5. The notary transfers the carbon coins from its wallet to the carbon wallet of microgrid *i*.

## 5. Case Studies

To verify the performance of local electricity and carbon trading between multi-energy microgrids, experiments are conducted in this section on a microgrid network consisting of three interconnected microgrids. All simulations are conducted in Matlab.

### 5.1. Scenario Description

**Figure 6:**
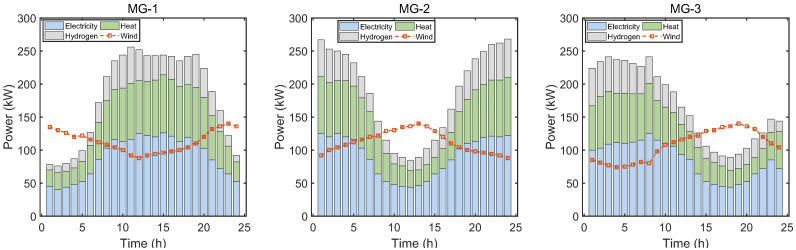
Load demands and wind power generation of microgrids.

**Figure 7:**
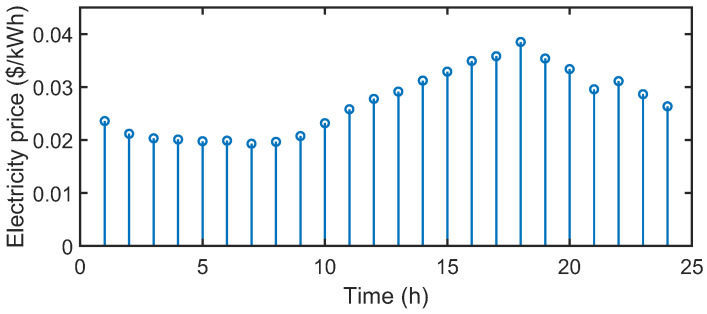
The electricity prices of the electric utility.

The test system is shown in Figure 2. Each microgrid has the same structures and energy devices. The optimization horizon is one day, and each time slot Δt is equal to one hour. Figure 6 depicts the electricity, heat, and hydrogen demands and wind power generation of microgrids. Figure 7 presents the electricity prices of the electric utility, which are obtained from the PJM database [26]. The gas prices are constant in all time slots, and are equal to 0.0571 $/kW [8]. The buying and selling prices of carbon allowance in the central carbon market are 0.06 $/kg and 0.02 $/kg, respectively [15]. The maximum amounts of electricity and gas that microgrids can purchase from the utilities in each time slot are both set as 1000 kW. Other parameters related to microgrids are tabulated in Table 1. Moreover, we consider that one electricity coin can be exchanged for USD 1 and one carbon coin can be exchanged for USD 2. That is, one carbon coin can be exchanged for two electricity coins.

### 5.2. Local Electricity Trading

**Figure 8:**
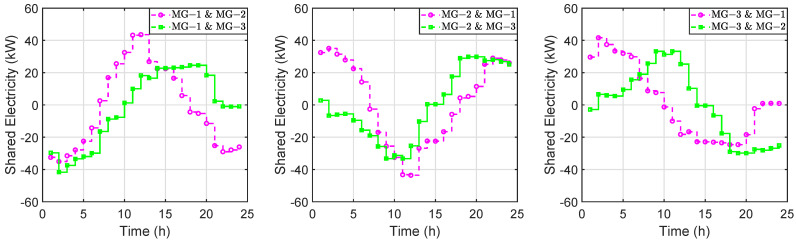
The electricity trading profiles between microgrids.

Figure 8 depicts the electricity trading profiles between microgrids. In Figure 8, if the shared electricity between microgrid *i* and *j* is a positive value, it means that microgrid *i* will purchase electricity from microgrid *j*; otherwise, if the shared electricity between microgrid *i* and *j* is a negative value, it means that microgrid *i* will sell electricity to microgrid *j*. It can be seen that microgrid 1 can obtain electricity in the daytime because of its high load demands but low wind power output during that period. Moreover, microgrid 2 will transmit electricity to the other microgrids during the day and microgrid 3 will supply electricity to other microgrids from time slots 10 to 24. This demonstrates that each microgrid actively participates in local electricity trading and acts as an electricity buyer or seller in different time slots.

### 5.3. Local Carbon Trading

**Figure 9:**
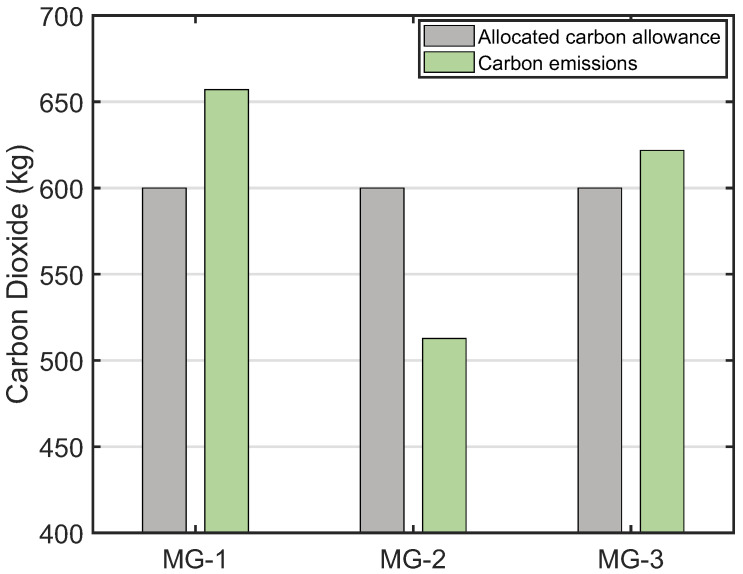
The allocated carbon allowance and carbon emissions of microgrids.

Figure 9 shows the initial carbon allowance and carbon emissions of microgrids. It can be seen that the carbon emissions of microgrids 1 and 3 exceed their allocated carbon allowance, but the carbon emissions of microgrid 2 are lower than its allocated carbon allowance. Specifically, microgrids 1 and 3 should purchase another 57.0121 kg and 21.7490 kg of carbon allowance, respectively. Microgrid 2 can sell 87.2163 kg of carbon allowance in the central or local carbon markets. Through local carbon trading, microgrids 1 and 3 can receive a total of 78.7611 kg of carbon allowance from microgrid 2. Therefore, microgrids 1 and 3 will not purchase carbon allowance from the central carbon market, while microgrid 2 will sell the remaining 8.4552 kg of carbon allowance in the central carbon market. Since the buying price of carbon allowance in the central carbon market is higher than the selling price, we know that local carbon trading can lower the total carbon cost of microgrids.

### 5.4. Electricity and Carbon Trading Payments

**Figure 10:**
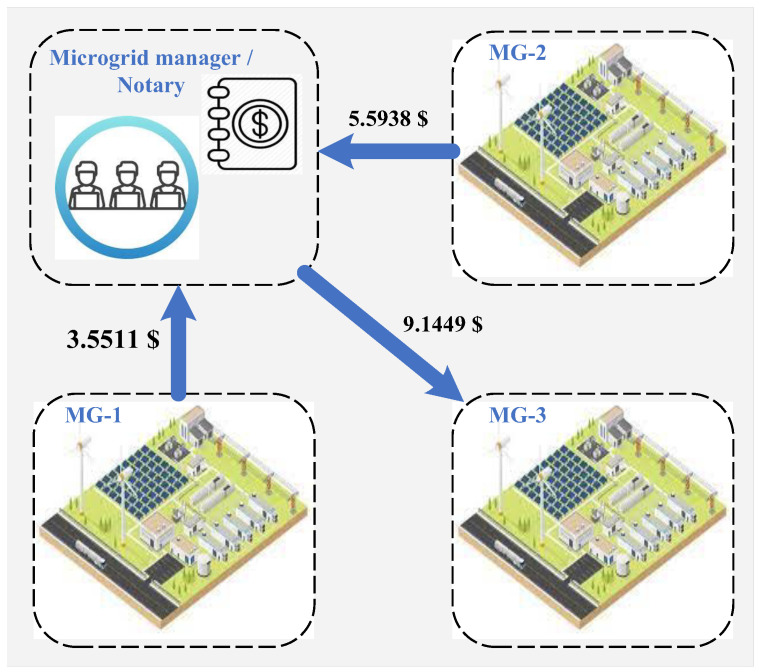
Local electricity market clearing results.

Figure 10 presents the clearing results of the local electricity market. The net electricity trading payments of microgrids are shown in the figure. According to the Nash bargaining solutions of the local electricity trading problem, the net electricity trading payments of microgrids 1 and 2 are 3.5511$ and 5.5938$, respectively. The net electricity trading payment of microgrid 3 is −9.1449$, which means that microgrid 3 will receive the revenue by sharing its electricity with other microgrids. This is because microgrid 3 mainly shares its electricity with other microgrids when the electricity prices are relatively high. Thus, microgrid 3 makes the greatest contribution in lowering the payment for microgrids.

**Figure 11:**
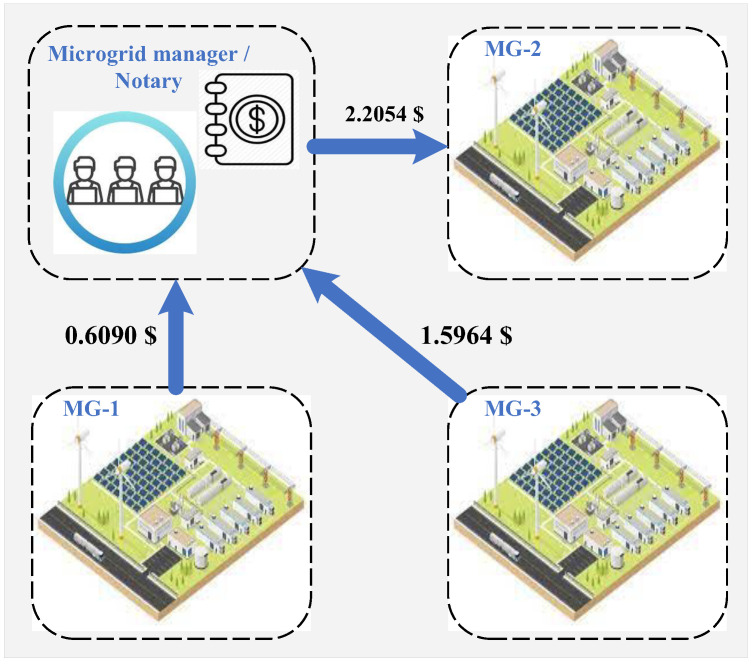
Local carbon market clearing results.

Figure 11 presents the clearing results of the local carbon market. The net carbon trading payments of microgrids are presented in the figure. It can be seen that microgrids 1 and 3 should pay for their received carbon allowance, and their corresponding payments are 0.6090$ and 1.5964$, respectively. Microgrid 2 shares its excess carbon allowance with other microgrids. Thus, it will receive 2.2054$.

### 5.5. Cross-Chain Interaction Results

As shown in Figure 10 and Figure 11, microgrid 2 will make a payment in the local electricity market but will receive a payoff in the local carbon market. Microgrid 3 will receive a payment in the local electricity market but will make a payment in the local carbon market. Thus, microgrids 2 and 3 can conduct the cross-chain interactions between the electricity blockchain and carbon blockchain.

After the implementation of local electricity and carbon trading, the microgrid manager will record the information of local electricity and carbon trading into the blockchains. Then, the cross-chain interactions will be conducted. Through local electricity and carbon trading, microgrid 2 will obtain 1.1027 carbon coins and will pay out 5.5938 electricity coins, while microgrid 3 will receive 9.1449 electricity coins and will pay out 0.7982 carbon coins. Thus, microgrid 2 can exchange all of its carbon coins into 2.2054 electricity coins, which will be used to pay for the obtained electricity. In the meantime, microgrid 3 can exchange 1.5964 electricity coins into carbon coins, which will be used to pay for the obtained carbon allowance. It is the cross-chain interactions that enables the value transfer between the electricity blockchain and carbon blockchain.

### 5.6. Comparison Results under Different Market Settings

To demonstrate the performance of the proposed market settings in lowering payments and carbon emissions for microgrids, four different market settings are compared, as shown in Table 2.

**Figure 12:**
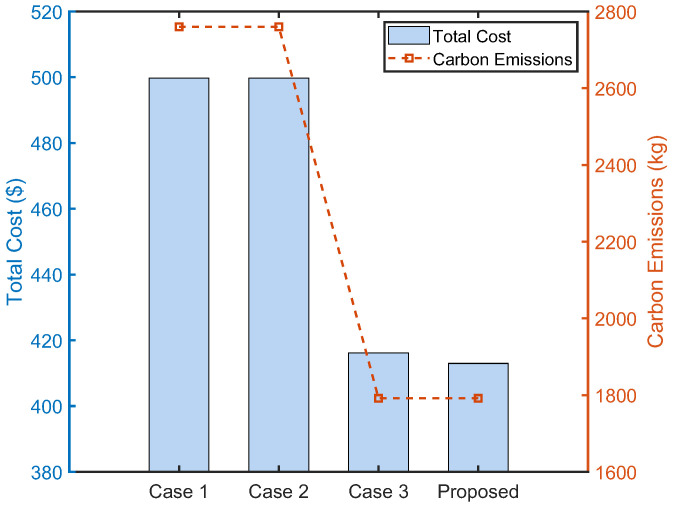
Comparison results under different market settings.

Figure 12 shows the comparison results of total costs and carbon emissions of microgrids under different market settings. It can be observed that under the proposed market setting, microgrids will have the lowest total cost and carbon emissions. Specifically, compared to Case 1, i.e., non-cooperative benchmarks, the total cost of microgrids decreases by 17.35% and the total carbon emissions of microgrids decrease by 35.09%. It can be explained that (i) local electricity trading allows microgrids to use more renewable energy while reducing the procurement of electricity; (ii) local carbon trading enables microgrids to lower the procurement of carbon allowance.

## 6. Conclusions

In this paper, we propose a local electricity and carbon trading method for multi-energy microgrids. The local electricity and carbon trading problems are formulated using the Nash bargaining theory. We solve the Nash bargaining problems by decomposing each problem into an energy/carbon scheduling problem and a payment bargaining problem. In addition, blockchain technology is used to record the transaction data and realize secure information interactions. A notary-mechanism-based cross-chain interaction method is proposed, achieving the value transfer between the electricity blockchain and carbon blockchain. The simulation results show that the proposed method is economically and environmentally beneficial for microgrids. Compared to the non-cooperative benchmarks, 17.35% of total cost and 35.09% of carbon emissions are reduced for microgrids. In future research, we will explore cross-chain interactions between electricity and carbon blockchains using other mechanisms, e.g., side chain and Hash locking.

## Figures and Tables

**Table 1:** Parameters for the microgrids (∀i∈M,∀STO∈S).

Parameter	Value	Unit	Parameter	Value	Unit
$R_{i}^{CHP,d}/R_{i}^{CHP,u}$	−20/20	kW/h	$P_{i,\max}^{ES,c}/P_{i,\max}^{HS,c}/P_{i,\max}^{H_{2}S,c}$	20/18/20	kW
$P_{i,\max}^{CHP,g}/P_{i,\max}^{GB,g}$	100/60	kW	$P_{i,\max}^{ES,d}/P_{i,\max}^{HS,d}/P_{i,\max}^{H_{2}S,d}$	20/18/20	kW
$P_{i,\max}^{ELE,h_{2}}/P_{i,\max}^{HFC,e}$	100/80	kW	$E_{i,\max}^{ES}/E_{i,\max}^{HS}/E_{i,\max}^{H_{2}S}$	30/25/30	kWh
$\eta_{i}^{CHP,e}/\eta_{i}^{CHP,h}$	0.32/0.408	-	$E_{i,\min}^{ES}/E_{i,\min}^{HS}/E_{i,\min}^{H_{2}S}$	6/5/6	kWh
$\eta_{i}^{STO,c}/\eta_{i}^{STO,d}$	0.95/0.95	-	$W_{i}^{CO_{2},a}$	600	kg
$\eta_{i}^{GB,h}/\eta_{i}^{ELE,h_{2}}/\eta_{i}^{HFC,e}$	0.7/0.87/0.85	-	$W_{i,max}^{CO_{2},buy}/W_{i,max}^{CO_{2},sell}$	500/500	kg
$em_{i}^{GRID}$	0.92	kg/kWh	$em_{i}^{CHP}$	0.202	kg/kWh
$em_{i}^{GB}$	0.202	kg/kWh	$em_{i}^{ELE}$	0.12	kg/kWh
$em_{i}^{HFC}$	0.15	kg/kWh	$em_{i}^{ES}$	0.083	kg/kWh

**Table 2:** Local market settings (Y: Yes; N: No).

Market Settings	Local Electricity Trading	Local Carbon Trading
Case 1	N	N
Case 2	N	Y
Case 3	Y	N
Case 4	Y	Y

## Data Availability

Not applicable.
